# Draft genome sequence of *Thermoanaerobacterium* sp. strain PSU-2 isolated from thermophilic hydrogen producing reactor

**DOI:** 10.1016/j.gdata.2017.02.012

**Published:** 2017-02-28

**Authors:** Sompong O-Thong, Peerawat Khongkliang, Chonticha Mamimin, Apinya Singkhala, Poonsuk Prasertsan, Nils-Kåre Birkeland

**Affiliations:** a Research Center in Energy and Environment, Faculty of Science, Thaksin University, Phatthalung 93210, Thailand; b Biotechnology Program, Department of Biology, Faculty of Science, Thaksin University, Phatthalung 93210, Thailand; c Department of Industrial Biotechnology, Faculty of Agro-Industry, Prince of Songkla University, Songkhla 90112, Thailand; d Department of Biology and Centre for Geobiology, University of Bergen, P.O. Box 7800, N-5020 Bergen, Norway

**Keywords:** Whole genome sequencing, *Thermoanaerobacterium* sp., Hydrogen producing bacteria, Thermophile

## Abstract

*Thermoanaerobacterium* sp. strain PSU-2 was isolated from thermophilic hydrogen producing reactor and subjected to draft genome sequencing on 454 pyrosequencing and annotated on RAST. The draft genome sequence of strain PSU-2 contains 2,552,497 bases with an estimated G + C content of 35.2%, 2555 CDS, 8 rRNAs and 57 tRNAs. The strain had a number of genes responsible for carbohydrates metabolic, amino acids and derivatives, and protein metabolism of 17.7%, 14.39% and 9.81%, respectively. Strain PSU-2 also had gene responsible for hydrogen biosynthesis as well as the genes related to Ni-Fe hydrogenase. Comparative genomic analysis indicates strain PSU-2 shares about 94% genome sequence similarity with *Thermoanaerobacterium xylanolyticum* LX-11. The nucleotide sequence of this draft genome was deposited into DDBJ/ENA/GenBank under the accession MSQD00000000.

**Table:** 

Specifications	
Organism/cell line/tissue	*Thermoanaerobacterium* sp.
Strain	PSU-2
Sequencer or array type	454 pyrosequencing
Data format	analyzed
Experimental factors	microbial strain
Experimental features	draft genome analysis and gene annotation of PSU-2
Consent	N/A
Sample source location	Songkhla, Thailand

## 1 Direct link to deposited data

The draft genome sequences could be found at the site http://www.ncbi.nlm.nih.gov/nuccore/MSQD00000000

## 2 Experimental design, materials and methods

The genus *Thermoanaerobacterium* is a group of anaerobic, gram-positive, rod shaped, reduce thiosulfate to elemental sulfur that belong to *Firmicutes* as previously described by Lee et al. [1]. Genus *Thermoanaerobacterium* are thermophilic that specialize in polysaccharide and carbohydrate fermentation, producing primarily L-lactic acid, acetic acid, ethanol, CO_2_, and H_2_ [2], [3]. The majority of characterized *Thermoanaerobacterium* strains have been isolated from hot springs and other thermal environments [4]; however, they have also been isolated from leachate of a waste pile from a canning factory [5], thermophilic bioreactor for biohydrogen production [6], [7] and deep subsurface environments [8]. This genus has been considered for biotechnological applications, such as conversion of lignocellulosic biomass to ethanol [3], biohydrogen and other chemicals [9]. *Thermoanaerobacterium* strain PSU-2 is a rod shaped, gram-positive, spore-forming and thermophilic hydrogen producing bacteria belonging to *Firmicutes* that was isolated from a biohydrogen reactor fed with palm oil mill effluent (POME). Phylogenetic analysis based on 16S rRNA genes indicated that strain PSU-2 belonged to the genus *Thermoanaerobacterium* [6]. This genus had been previously studied for hydrogen production from various carbohydrates, such as starch, sucrose and molasses [10]. Strain PSU-2 has a high hydrogen production capacity within a wide range of pH (4.5–8) and temperature (45–70 °C), with the optimal temperature 60 °C and optimal initial pH about 6.25. The strain performed ethanol–acetate type fermentation in inorganic nitrogen amended medium, while it performed butyrate–acetate type fermentation in organic nitrogen amended medium [6].

The draft genome of strain PSU-2 was sequenced with 454 technology using a GS-FLX pyrosequencer at GATC Biotech, Germany (http://www.gatc-biotech.com). A total of 2,552,497 bases were obtained. Assembly into 44 contigs was done with Newbler version 2.9 accessed through the Lifeportal, University of Oslo (http://www.uio.no/english /services/it/research/hpc/lifeportal) and annotation was conducted on RAST [11]. SEED viewer was used for subsystem functional categorization of the predicted open reading frames (ORFs) and visualization [12]. An average nucleotide identity (ANI) was analysis using the online ANI calculator (http://enve-omics.ce.gatech.edu/ani/index). An *In Silico* genomic DNA:DNA hybridization was performed by genome-to-genome distance calculator (http://ggdc.dsmz.de).

## 3 Data description

**Fig. 1:**
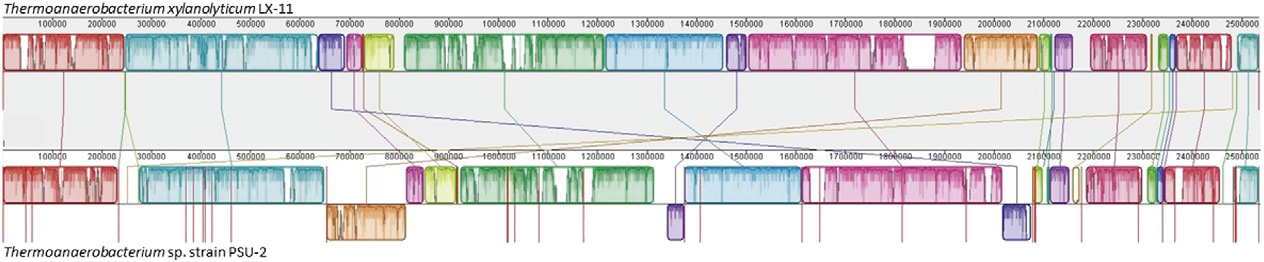
Draft genome alignment of *Thermoanaerobacterium* sp. strain PSU-2 with *Thermoanaerobacterium xylanolyticum* LX-11.

**Fig. 2:**
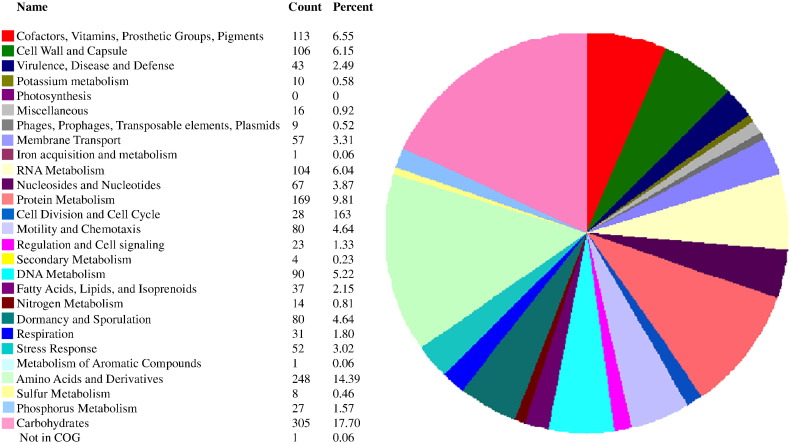
Distribution and counts of genes in COG categories for draft genome of *Thermoanaerobacterium* sp. strain PSU-2.

The draft genome of *Thermoanaerobacterium* sp. strain PSU-2 consisted of single DNA chromosome of 2,552,497 bases, a G + C content of 35.2%. The draft genome was predicted to contain 2555 protein-coding sequence, 8 rRNAs and 57 tRNAs. These genes were annotated and classified into 337 subsystems. Most of the annotated genes were involved in carbohydrates metabolic (17.7%), amino acids (14.39%) and derivatives, and protein metabolism (9.81%) (Fig.1). Strain PSU-2 also had gene responsible for hydrogen biosynthesis as well as the genes related to Ni-Fe hydrogenase. Comparative genomic analysis indicates PSU-2 by an average nucleotide identity (ANI) analysis using the online ANI calculator (http://enve-omics.ce.gatech.edu/ani/index) revealed ANI values of 94, when the PSU-2 draft sequence was compared with complete sequences of *Thermoanaerobacterium xylanolyticum* LX-11 species, isolated from geothermal areas of Yellowstone National Park, Wyoming, USA (Fig. 2). This indicates that PSU-2 represents a separate species, as this value is lower than the threshold value of 95%, which corresponds to a genomic DNA:DNA hybridization value of 70% and is a common threshold value for distinction between species [13].

## 4 Nucleotide accession number

This whole genome project has been deposited at DDBJ/ENA/ GenBank under accession no. MSQD00000000. The version described in this paper is version MSQD01000000.

## Conflict of interest

The authors clarified that this work and writing has no conflict of interest.
